# Assessing the knowledge, attitude and perception on workplace readiness regarding COVID-19 among health care providers in Ethiopia—An internet-based survey

**DOI:** 10.1371/journal.pone.0247848

**Published:** 2021-03-04

**Authors:** Agazi Fitsum Gebreselassie, Abebe Bekele, Heaven Yeshaneh Tatere, Rex Wong

**Affiliations:** 1 University of Global Health Equity, Kigali, Rwanda; 2 Addis Ababa University, Addis Ababa, Ethiopia; 3 Yale University, New Haven, Connecticut, United States of America; 1. IRCCS Neuromed 2. Doctors with Africa CUAMM, ITALY

## Abstract

**Background:**

Healthcare facilities in Ethiopia are responsible for collecting samples for testing and treating COVID-19 patients, providing COVID-19 information to staff, establishment of response teams, and provision of adequate personal protective equipment (PPE). Working at the frontlines against the pandemic, health care providers’ level of knowledge about COVID-19, attitude towards their work, and confidence in the preparedness of their facilities are essential factors in mounting a successful response.

**Objectives:**

This study investigated the knowledge level of HCP in Ethiopia on this novel coronavirus, and their perspectives on whether their workplaces have sufficient preparedness to handle this disease.

**Methods:**

A self-administered online survey was conducted.

**Results:**

The knowledge related to COVID-19 among HCPs was high, with an overall average of 91.5%. The majority of our respondents were supportive to the government’s measures to minimize disease transmission, but most of them were also frustrated by how COVID affected their day to day lives. The majority of them were worried about contracting COVID at work and transmitting the infection to their families. Most respondents did not feel safe going to work (P<0.001). Apart from providing adequate information on COVID-19, most workplaces did not have sufficient PPE (P<000.1) and medical supplies (P<0.001). Close to 50% of respondents agreed and disagreed that their workplaces had clear protocols for handling COVID-19 (P = 0.144). Those who handled known COVID patients were more likely to agree their workplaces had clear protocols (OR = 2.69, P<0.001).

**Conclusion:**

Improving supplies of PPEs and establishing a clear communicating protocol in handling COVID patients are highly recommended.

## Introduction

The Novel Coronavirus, the cause of a new respiratory infectious disease (COVID-19) belongs to a big virus’s family causing diseases that resembles the common cold to Severe Acute Respiratory Syndrome (SARS) and Middle East Respiratory Syndrome (MERS) [1,2]. Since first discovered in Wuhan City, China in December 2019, the disease has become a pandemic as declared by WHO on 11 March 2020 and affected over 3 Million people in 210 countries [3,4].

The common COVID-19 symptoms include shortness of breathing, coughing, and difficulties in breathing [4]. It is a highly contagious and deadly disease, with a mortality rate of about 7% [5]. The transmission of COVID-19 from humans to humans is mainly reported through droplets via sneezing or coughing and contact with surfaces touched by the infected person [2]. As of today, there is no vaccine available for the disease [2]. Since health care providers (HCP) are working in an environment that exposes them to potential infected persons, they are at a higher risk of being infected [6,7]. Many HCPs around the world have lost their lives due to COVID-19 [8–10].

One of the countries battling the epidemic is Ethiopia. The country’s healthcare system is structured along three tiers: primary, secondary, and tertiary [11]. The primary level consists of health posts, health centers, and primary hospitals with the capacity to serve from 3,000 to 100,000 people. The secondary and tertiary levels are made up of general and referral hospitals respectively, with the capacity to serve one to five million people. By 2017, the country had a total of 118,507 working healthcare professionals, translating to 1.26 healthcare providers per 1000 population [12]. Since the first confirmed case of COVID-19 in Ethiopia on 13 March 2020, Ethiopia has over 6900 confirmed cases and 89 deaths as of July 2020; translating to a mortality rate of 1.7% [13].

The country has implemented a series of measures in response to the COVID-19 outbreak. For example, flights to over 80 countries were suspended, the Ministry of Health started contact tracing and public education campaigns [14]. The government also closed down schools (medical schools included), started restricting large public gatherings and the movement of people within the country, and locked down a few areas in the capital [15]. Health care services at hospitals, health centers and health posts continue to provide services to the public, although the services have been significantly affected–elective surgeries were cancelled, emergency and facility deliveries have been reduced, and some convention venues had been converted to treatment sites [14,15]. Healthcare facilities are responsible for providing COVID-19 information to staff, establishment of response teams, and provision of adequate personal protective equipment (PPE) [16]. Ultimately, working in the frontlines against the pandemic, HCP’s level of knowledge about COVID-19, attitude towards their work, and confidence in the preparedness of their facilities are essential factors in mounting a successful response. Accordingly, this study aims to investigate the knowledge level of HCP in Ethiopia on this novel coronavirus, and their perspectives on whether their work places have sufficient preparedness to handle this disease.

## Methods

### Study design and setting

A cross sectional study was conducted to collect the information from health care professionals practicing in Ethiopia from May to June 2020.

### Study sample

Our sample consisted of HCPs in Ethiopia–including physicians, dentists, nurses, pharmacists, physiotherapists, lab technicians, ophthalmologists and others working in public or private hospitals, health centers, and health posts. Those who have been working in any of the health facilities in the country since Jan 2020 were included in the study to ensure the HCPs participated in the study were not new to the health facility. Given there are over 20,000 HCPs in Ethiopia with internet penetration of 20%, [17] and assuming we could reach 50% of the HCPs who have access to the internet and a 20% response rate, we estimated our target sample size to be about 400.

### Study instrument

A self-administered, 34-question online survey with 4 major sections was used to collect data. Section one collected basic demographic information. Section two had 12 closed ended questions assessing HCPs’ knowledge about COVID-19. Each question had the answer option of “yes” or “no”. Section three had 12 questions related to the respondents’ attitude on COVID-19 and section four had 10 questions about their perception on the readiness of their workplace for dealing with COVID-19. Both section three and four used 4-point Likert-scale statements, with the options of “strongly agree”, “agree”, “disagree” and “strongly disagree”. The tool was developed based on COVID-19 online information available from the World Health Organization [2] and Centers for Diseases Control [1]. The tool was in both English and Amharic and was translated and back translated and was pretested on 10 HCPs in Ethiopia before being finalized.

### Data collection method

An online survey was used to collect the data between 13 May and 6 June 2020. The survey link was shared through social media and was emailed to individuals as well as professional associations in the country.

The first page of the online survey provides the information of the study. Participants were required to submit an online consent by clicking on a button before directed to the survey.

### Ethical approval

The study was approved by the Internal Review Board at the University of Global Health Equity (protocol # 0110).

### Measures

Three key measures were included in this study:

1. Knowledge level of COVID-19
2. Attitude toward COVID-19
3. Perspectives about workplace readiness for COVID-19

### Data management and analysis

Descriptive analyses were conducted for demographic information. The knowledge score was calculated as the percentage of correct answers divided by questions answered. The overall knowledge was categorized as “good” if the score was between 80 and 100%, moderate to poor if less than 80% [18].

One-sample binomial tests were conducted to detect the significance for statements with 4-point Likert scale, by comparing the percentage of choosing “agree” and “strongly agree” to the percentage of choosing “disagree” and “strongly disagree”, with tested value set at 50% with the assumption of equal chance of selection [19].

Fisher Exact tests were used to analyze the association between the key measures by demographics. All analyses were conducted using SPSS v. 23 (IBM) with P-value set at 0.05.

## Results

A total 400 people visited the survey link. Two did not consent. Out of the 398 consented, 52 did not meet the selection criteria—9 were not health care providers, and 43 had not been working in any healthcare facilities since January 2020. Out of the 346 who fulfilled the selection criteria, three did not complete the survey. Resulting in a final sample size of 343.

The majority of our respondents were physicians (n = 266, 77.6%), followed by nurses and midwives (n = 29, 8.5%). Out of the total 343 respondents, 248 (73.2%) were male, 225 (68.2%) were below the age of 30 years, 249 (72.5%) were single, 91 (26.5%) were married (Table 1).

**Table 1:** Demographics.

		N (%)
Sample		343
Profession	Physician	266 (77.6%)
	Nurse and Midwife	29 (8.5%)
	Dentist	14 (4.1%)
	Health officer	11 (3.2%)
	Others*	22 (6.4%)
Gender	Female	90 (26.8%)
	Male	248 (73.2%)
Age group	Below 30 years	225 (68.2%)
	30 years or above	105 (31.8%)
Education	Diploma/Bachelor	66 (19.4%)
	Master	12 (3.5%)
	MD or PhD	263 (77.1%)
Facility sector	Public	315 (92.4%)
	Private	26 (7.6%)
Facility type	Health center	12 (3.5%)
	Primary/General/Private hospital	129 (37.8%)
	Referral hospital	200 (58.7%)
Work experience (years)	Less than 5 years	252 (73.5%)
	5 to 10 years	75 (21.9%)
	More than 10 years	16 (4.7%)
Received, screened, or treated any known COVID patient	No	266 (77.8%)
	Yes	76 (22.2%)
Marital status	Single/	249 (72.5%)
	Divorced/widowed	3 (1%)
	Married or cohabitate	91 (26.5%)
Source of COVID information	News	195 (56.9%)
	Social media	224 (65.3%)
	Family and friends	73 (21.3%)
	Scientific journal or website	262 (76.4%)
	Other healthcare professionals	154 (44.9%)

* Environmental health professional, anesthetist, medical imaging, ophthalmologist, medical intern, pharmacist, psychiatry professional, public health specialist.

Most of the respondents were working in the public sector (n = 315, 92.4%). More than half of the respondents (n = 200, 58.7%) worked in referral hospitals, 252 (73.5%) had less than 5 years of work experience, and only 76 (22.2%) had received, screened or treated any known COVID patients (Table 1).

The most common sources of COVID information were scientific journals or websites (n = 262, 76.1%), social media (n = 224, 65.3%) and the mainstream news media (n = 195, 56.6%) (Table 1).

The overall knowledge score was 91.45% (SD ±8.1%), with 328 (95.9%) scoring 80% or above. The question with the lowest score was: “wet cough is a possible symptom of COVID-19” (40.9%) (Table 2).

**Table 2:** HCPs’ knowledge about COVID-19.

Knowledge on COVID-19		Correct
COVID-19 is transmitted by close contact with an infected person		99.7%
Fever is a possible symptom of COVID-19		99.4%
Wet cough is a possible symptom of COVID-19		40.9%
Shortness of breath is a possible symptom of COVID-19		99.4%
Watery diarrhea is a possible symptom of COVID-19		79.1%
The COVID-19 incubation period is between 2–14 days		98.5%
COVID-19 vaccine is available		95.6%
Antibiotics is the first-line treatment		87.9%
Washing hands with soap and water can help in the prevention of disease transmission		99.7%
The disease is more dangerous for people with weak immune system		98.8%
The disease can be transmitted through contact with infected surfaces		98.8%
The disease is more dangerous for people with chronic diseases		98.8%
Overall knowledge score	Mean (SD)	91.5% (±8.1%)
	Less than 80%	14 (4.1%)
	80% or more	328 (95.%)

Significantly higher percentage of respondents worried about contracting the virus due to work (n = 325, 96%, P<0.001), and worried about their families contracting the illness because of their work (n = 307, 90%, P<0.001) (Table 3). Most of the respondents (n = 307, 90%) said they were frustrated with how COVID was affecting their daily lives (p<0.001). Majority of the respondents (n = 208, 82%) stated that their families were supportive of their work, while 325 (95%) of the respondents said they supported the government’s travel restrictions (p<0.001) (Table 3).

**Table 3:** Attitude and workplace preparedness*.

**Attitude about COVID-19**	Strongly agree/agree	Strongly disagree/disagree	P-value
I am worried that I will get COVID-19 due to work	326 (96%)	15 (4%)	<0.001
I am worried that my family members may get COVID-19 due to my work	308 (90%)	35 (10%)	<0.001
I am frustrated about how COVID-19 is affecting my daily life	308 (90%)	33 (10%)	<0.001
My family is supportive for me to continue to work at the health facility	281 (82%)	61 (18%)	<0.001
I support the government in restricting travel to control COVID-19 transmission	326 (95%)	17 (5%)	<0.001
**Workplace preparedness**	Strongly agree/agree	Strongly disagree/disagree	p-value
My workplace provides me enough information about COVID-19	218 (64%)	124 (36%)	<0.001
My workplace has a clear protocol for handling COVID-19	185 (54%)	157 (46%)	0.144
I feel safe going to work everyday	143 (42%)	198 (58%)	0.003
My workplace has sufficient PPE to handle COVID-19 patients	131 (38%)	210 (62%)	<0.001
My workplace has sufficient medical supplies to handle COVID-19 patients	127 (37%)	213 (63%)	<0.001

*The sum of respondents for each question might not be equal to the total sample size of 343 as some respondents have elected not to respond to all the questions.

For the five questions related to workplace preparedness, significantly more respondents (n = 218, 64%) said their workplace provided enough information about COVID-19 (P<0.001). Statistically, the proportions of respondents who agreed that their workplace had a clear protocol for handling COVID-19 (n = 184, 54%) was no different than the proportions of respondents who disagreed (n = 157, 46%, P = 0.144). Significantly higher percentage of respondents indicated that their workplace didn’t have sufficient PPE (n = 210, 62%, P<0.001), didn’t have sufficient medical supplies to handle COVID patients (n = 213, 63%, P<0.001) and they didn’t feel safe going to work everyday (n = 198, 58%, P = 0.003) (Table 3).

Further analysis showed respondents who had received, screened, or treated any known COVID patients had 2.69 higher odds to agree that their workplaces have clear COVID handling protocol (P<0.001). Whether private or public hospital (P = 0.306), or the type of health care facility (0.07) did not show any significant association to the workplace COVID handling protocol (Table 4).

**Table 4:** Factors associated with HCPs’ perceptions about their workplace having clear COVID handling protocol^+^.

Workplace has clear COVID handling protocol		Disagree	Agree	Unadjusted OR	P-value
Facility sector	Public	149 (94.3%)	166 (90.7%)	NA	0.306
	Private	9 (5.7%)	17 (9.3%)		
Facility type	Health center	3 (1.9%)	9 (4.9%)	NA	0.07
	Primary/general/private hospital	52 (33.3%)	76 (41.3%)		
	Referral hospital	101 (64.7%)	99 (53.8%)		
Received, screened, or treated any known COVID patient	No	136 (86.6%)	130 (70.7%)	2.69	<0.001*
	Yes	21 (13.4%)	54 (29.3%)		

*significant at P = 0.05.

^+^The sum of respondents for each question might not be equal to the total sample size of 343 as some respondents have elected not to respond to all the questions.

## Discussion

Since the first case was reported in China in December 2019, COVID-19 has had devastating effects globally. Since the first confirmed case in Ethiopia in March 2020, there have been over six thousand cases. To our knowledge, this study is the first in studying the knowledge, attitudes and the perception of workplace readiness of health care providers in Ethiopia. Since health care providers are at the frontline of handling COVID-19, it is important to have this information to guide the decision-making in health facility preparedness, in order to protect both the public and the health care providers.

The study results indicated the knowledge related to COVID-19 among HCPs was relatively high, with an overall average of 91.5%, and over 95% of respondents scored higher than 80%, indicating the health care providers in Ethiopia were well informed about COVID. Some other studies on health care providers’ knowledge on COVID in Rwanda and Uganda showed similar findings [20,21]. In our study, most respondents were physicians (77%), and many of them acquired COVID information from scientific websites (76%). Since the WHO, CDC, Ministry of Health, EPHA official website have clearly posted the most updated COVID information on a daily basis, there is no shortage of information about COVID. Respondents in our study have access to the internet, it was reasonable to assume that they could acquire the most up-to-date information about COVID from such websites.

Since there is no effective cure for COVID at the moment, social distancing, vigorous public health practices including wearing masks, proper hand washing, proper coughing and sneezing techniques remain the most important measures to combat against COVID. Such course of actions required the understanding and cooperation of the public, and, without a doubt, significantly affected all lives. Our study results reflected such circumstances. While the vast majority of our respondents were supportive to the government’s restriction in travel as a measure to minimize disease transmission, most of them were also frustrated by how COVID affected their day to day lives.

The majority of them were worried about contracting COVID at work as well as worried about their families being infected due to their work. Healthcare providers are working at the frontline dealing with a highly contagious and deadly disease, with no effective vaccine. Their concerns and worries were understandable.

There was no statistical difference in the perceptions of workplace readiness between private and public hospitals. Based on the study results, most respondents did not feel their workplaces were ready for handling COVID. Apart from providing adequate information on COVID-19, most workplaces did not have sufficient PPE (P<0.001) and medical supplies (P<0.001), and most respondents did not feel safe going to work (P<0.001).

There were no statistical differences in the proportions of respondents who agreed and disagreed that their workplaces had clear protocols for handling COVID-19 (P = 0.144). However, those who handled known COVID patients were more likely to agree their workplaces had clear protocols (OR = 2.69, P<0.001). In Ethiopia, the Ethiopian Federal Ministry of Health has developed and provided the national COVID-19 guideline to all health facilities. However, a health facility having a COVID protocol does not translate into all employees understanding it. This result could potentially be due to COVID protocols not clearly communicated outside those who had to handle COVID patients. Further studies on this is needed.

The state of medical supplies even prior to the onset of the pandemic was weak at best, with only 557 ventilators available in a country of more than 100 million people [22], and stock out of many essential medicine were prevalent [23–25]. Much like the rest of the world, the main strategy employed by the country for fighting the pandemic has been focusing on promoting social distancing and handwashing and not necessarily strengthening the medical supply [26]. Despite the Ethiopian government had set up a fund to distribute masks and sanitizers to the public since the outbreak started [27] and some industrial private firms have switched to producing masks and other PPE [15], these materials haven’t necessarily made it to the healthcare facilities. Indeed, the inadequate supply of PPEs was reflected in the number of HCPs infected by the disease. As of June 23, 87 HCPs were infected, constituting 1.8% of the total 4848 confirmed cases [28]. This should not be taken lightly, especially in a country that is already grappling with human resource challenges. The results of this study suggested there is a need to strengthen the health system by improving supplies at health care facilities as well as establishing and communicating clear protocol in handling COVID patients.

Our findings must be interpreted in light of some limitations. First, our study used an online survey in data collection and the generalizability could be limited. This inevitably might have skewed the study results by only including respondents with access to the internet, which in turn, easier access to information. Given the access to internet and medical supplies outside urban Ethiopia has been historically challenging [29–32], we anticipate the actual knowledge level and workplace preparedness would be lower than our study results. Second, our study investigated the perceived workplace readiness. We could not confirm if the actual supply was aligned with the data in this self-administered survey. However, our sample did have representations of both private and public sectors, as well as all levels of health facilities.

Overall, findings from this survey suggest that the healthcare workers have a relatively high knowledge level about COVID-19. However, they were universally worried about the situation. Medical supplies and PPE to handle COVID need to be strengthened. As new information of COVID is emerging daily, most updated information should be communicated to all health care providers in a regular and transparent manner to ensure their confidence and reduce their stress level when handling COVID at the frontline. Interventions to reduce HCP’s worries and concerns about contracting COVID at work is necessary.
